# Perioperative Care and Clinical Outcomes of Patients with Left Ventricular Assist Devices Undergoing Noncardiac Surgery in Korea: A Retrospective Study

**DOI:** 10.3390/jcm15051748

**Published:** 2026-02-25

**Authors:** Yeonji Noh, Dahee Hyun, Dong-Jae Kim, Jong-Hwan Lee, Yang Hyun Cho, Jeong-Jin Min

**Affiliations:** 1 Department of Anesthesiology and Pain Medicine, Samsung Medical Center, Sungkyunkwan University School of Medicine, 81 Irwon-ro, Gangnam-gu, Seoul 06351, Republic of Korea; yeonji.noh@samsung.com (Y.N.); jongwhanlee75@gmail.com (J.-H.L.); 2 Department of Medicine, Sungkyunkwan University School of Medicine, Seoul 06351, Republic of Korea; 3 Department of Thoracic & Cardiovascular Surgery, Samsung Medical Center, Sungkyunkwan University School of Medicine, Seoul 06351, Republic of Korea; mdcho95@gmail.com

**Keywords:** LVAD, non-cardiac surgery, mortality, complications

## Abstract

**Background**: Since 2018, the number of left ventricular assist devices (LVAD) implantations in Korea has been steadily increasing. Consequently, an increasing number of LVAD patients are presenting for non-cardiac surgery (NCS) of varying complexity. However, recent data on the perioperative management and clinical course of these patients remain limited. We share our investigation on patient and perioperative risk factors, as well as perioperative adverse outcomes, including mortality, in LVAD patients undergoing NCS. **Methods**: We retrospectively reviewed medical records of 36 LVAD patients who underwent NCS at our tertiary care center between 2018 and 2024. Patients requiring VA-ECMO were excluded. The primary end point was in-hospital mortality. The secondary end point was a composite of complications, including postoperative pulmonary complications, acute kidney injury, cerebrovascular accident, postoperative bleeding or thrombosis, and hemodynamic instability. Using univariable and multivariable logistic regression analysis, we examined the correlation between perioperative factors and adverse outcomes. **Results**: A total of 53 NCS index cases across 40 hospitalizations were analyzed. General surgery was the most common specialty (*n* = 19, 35.8%), followed by thoracic surgery (*n* = 13, 24.5%), plastic surgery (*n* = 7, 13.2%), and neurosurgery (*n* = 4, 7.5%). Thirteen procedures (24.5%) were classified as major surgeries. Postoperative complications occurred in 24 patients (66.7%), and 8 patients (20%) experienced mortality. Multivariable regression analysis identified major surgery (adjusted odds ratio [aOR] 1.44; 95% CI 1.11–1.86; *p* = 0.010), and intraoperative transfusion of ≥3 units of packed red blood cells (aOR 1.47; 95% CI 1.05–2.04; *p* = 0.029) as significant predictors of in-hospital mortality. Undergoing NCS within 180 days after LVAD implantation was associated with an increased risk of composite complications (aOR 1.86; 95% CI 1.53–2.27; *p* < 0.001). **Conclusions**: LVAD patients undergoing non-cardiac surgery frequently experience postoperative complications. Major surgeries, significant intraoperative transfusions, and early surgery following LVAD implantation are key predictors of poor outcomes. Careful risk assessment and tailored perioperative management are essential in this population.

## 1. Introduction

Left Ventricular Assist Devices (LVADs) have become the standard treatment for patients with heart failure, serving as either a bridge to transplant or destination therapy. Since October 2018, when LVADs were approved for coverage under the Korean National Health Insurance, the number of patients receiving LVADs has been increasing [1].

Advancements in durable LVAD technologies have improved longevity, leading to a rise in the frequency of non-cardiac surgeries in LVAD patients, either due to LVAD-related complications or their comorbidities [2]. These patients are at high risk of intraoperative cardiovascular and cerebrovascular events and require continuous anticoagulation therapy, which not only increases the risk of complications but also serves as an indication for non-cardiac surgery [3]. The unique characteristics of both LVAD patients and the devices themselves pose significant challenges to anesthesiologists in managing intraoperative care. Therefore, understanding perioperative management, clinical outcomes, and related factors in LVAD patients is crucial for ensuring hemodynamic stability.

From 2012 to 2020, Korean patients primarily received the HeartMate (HM) II (Thoratec Corp., Pleasanton, CA, USA) and HeartWare VAD (HVAD; HeartWare Inc., Framingham, MA, USA), the latter of which was recalled in 2021 due to safety concerns [4]. In September 2020, the HeartMate (HM) 3 (Thoratec Corp., Pleasanton, CA, USA) was introduced, and it has since become the most commonly implanted LVAD in Korea.

Most previous studies on anesthetic management for non-cardiac surgery in LVAD patients predominantly included devices which are not used anymore, with limited data available on patients supported by newer centrifugal-flow LVADs, such as HM3. Our study specifically focuses on patients with predominantly third-generation centrifugal LVADs, providing valuable insights into perioperative management and outcomes in this contemporary patient population.

## 2. Methods

This study was approved by the Institutional Review Board of Samsung Medical Center (IRB No. SMC 2025-02-113-002, approved on 26 February 2025). The requirement for informed consent was waived due to the retrospective nature of the study.

### 2.1. Study Population

We conducted a single-center retrospective cohort study of LVAD patients who underwent non-cardiac surgery requiring an anesthesiologist after LVAD implantation and before heart transplantation between January 2018 and December 2024. While the specific anesthetic technique was determined by the attending anesthesiologist, surgical cases requiring anesthesiologist supervision beyond the scope of simple local infiltration anesthesia were identified and scheduled by the respective surgical departments. For patients who later underwent heart transplantation, only non-cardiac surgical procedures performed before heart transplantation were included in the analysis. Patients were excluded if they underwent non-cardiac surgery while in a veno-arterial extracorporeal membrane oxygenation (ECMO) state.

### 2.2. Data Collection

All data were extracted from the electronic health record, including demographics, preoperative evaluations, intraoperative records, and postoperative courses. To ensure accuracy and reliability, the data were reviewed multiple times by different investigators.

Non-cardiac surgery was defined as any surgical procedure that does not involve the heart or its major vessels. The classification of major surgery followed the criteria described by Martin et al. (2020) [5], including intraoperative factors such as vascular clamping or organ ischemia, significant blood loss (≥500 mL), high vasoactive–inotropic scores (≥5 points), and prolonged operative time (>4 h), as well as postoperative metabolic stress response.

Demographic and preoperative variables were selected based on previously published data [2,6,7] and clinical relevance. Specifically, the collected baseline characteristics included comorbidities, American Society of Anesthesiologists (ASA) classification, functional class, heart failure type, and the presence of an implantable cardioverter–defibrillator.

LVAD-related variables were specifically investigated for each patient, including LVAD type, time to implantation, duration of support, occurrence of any events or complications, and key parameters (flow, speed, pulsatility index, and power).

Preoperative evaluations included laboratory tests (hemoglobin, hematocrit, platelet count, lymphocyte count, electrolytes, creatinine, liver function tests, coagulation parameters, and cardiac enzymes), chest radiography results, and right heart function variables measured by transthoracic echocardiography. Preoperative anticoagulation and antiplatelet agents, preoperative vasopressor or inotropic support, and line status (arterial and central lines) were also assessed.

The intraoperative anesthetic records were reviewed for the type of surgery, anesthetic characteristics, anesthesia provider, intraoperative vasoactive medications, vital signs, hypotensive events requiring resuscitation, fluid administration, transfusion, estimated blood loss, use of invasive monitors, and discharge location.

Intraoperative hypotension was quantified using blood pressure data automatically recorded at 5 min intervals. The area for recorded mean blood pressure (MBP) values below 60 mmHg was calculated using the trapezoidal rule, with results expressed in mmHg × minutes. The time-weighted average (TWA) of MBP below the threshold of 60 mmHg was calculated by dividing the area for MBP <60 mmHg by the total monitoring time.

Postoperative records included laboratory results (creatinine, liver function tests, lactate dehydrogenase, coagulation parameters, C-reactive protein, and cardiac enzymes), any complications within one week, transfusion requirements, LVAD parameters (flow, speed, pulsatility index, and power), mortality, and hospital length of stay.

### 2.3. Outcomes

The primary endpoint was in-hospital all-cause mortality, assessed per hospitalization. For patients who underwent multiple surgeries during the same hospitalization, the index case was defined as the first operation, with priority given to major over minor procedures. The secondary endpoint was a composite of postoperative complications, including postoperative pulmonary complications (PPC), cerebrovascular accident (CVA), hemorrhage within postoperative 72 h, clinically significant hypotension requiring treatment, thrombotic events, and acute kidney injury (AKI). These complications were defined as new postoperative events rather than preexisting conditions. Definitions for PPC, CVA, thrombotic events, AKI, and postoperative hemorrhage were based on the European Perioperative Clinical Outcome (EPCO) criteria [8]. PPCs were defined as the occurrence of respiratory infection, respiratory failure, pleural effusion, atelectasis, pneumothorax, bronchospasm, or aspiration pneumonitis. CVA was defined as an embolic, thrombotic, or hemorrhagic cerebral event. Thrombotic events included deep vein thrombosis and pulmonary thromboembolism. AKI was defined as an increase in postoperative serum creatinine to more than 1.5 times the preoperative value or an absolute increase of at least 0.3 mg/dL within 7 days. Postoperative hemorrhage was defined as bleeding occurring within 72 h postoperatively, including gastrointestinal (GI) bleeding. Postoperative hypotension was defined as a mean blood pressure (MBP) of less than 60 mmHg requiring vasopressor support, fluid resuscitation, or transfusion [9]. To avoid duplication of composite outcomes and hemodynamic parameters, patients undergoing multiple procedures were limited to a single index case, defined as the earliest non-cardiac surgery evaluated within each 7-day period [6].

### 2.4. Statistical Analysis

Categorical variables were reported as frequencies and proportions, while continuous variables were expressed as mean ± standard deviation (SD) or median with interquartile range (IQR), as appropriate. Categorical variables and outcomes were compared using the Chi-squared test or Fisher’s exact test. Univariable logistic regression was performed to identify potential risk factors associated with in-hospital mortality or composite complications. Adjusted odds ratios (aORs) and 95% confidence intervals (CIs) were subsequently calculated using multivariable logistic regression. Candidate variables included age, comorbidities, functional status, type of surgery, intraoperative factors (e.g., bleeding, transfusion, vasopressor use), and duration since LVAD implantation. These variables were selected based on previously published data [2,6,7] and clinical relevance. The final model was then determined using backward selection with the lowest Akaike information criterion (AIC).

All statistical analyses were performed using R version 4.4.2 (R Foundation for Statistical Computing, Vienna, Austria), with a two-sided *p*-value < 0.05 considered statistically significant.

## 3. Results

**Figure 1:**
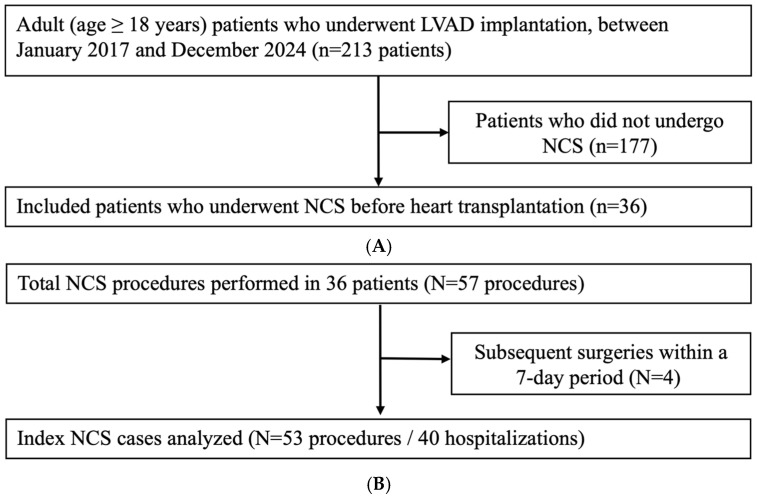
(**A**) Selection of study participants: From an initial screening of 213 patients who underwent LVAD implantation, 36 patients met the inclusion criteria for undergoing non-cardiac surgery (NCS). (**B**) Selection of procedures and hospitalizations: A total of 57 NCS procedures were identified from the included patients. After consolidating subsequent surgeries within a 7-day window, 53 index cases were identified for the analysis of composite complications. Furthermore, because mortality was assessed on a per-hospitalization basis, these procedures were categorized into 40 distinct hospitalizations for the mortality analysis. LVAD, Left ventricular assist device; NCS, Non-cardiac surgery.

A total of 36 LVAD patients were included, undergoing a total of 57 NCS procedures. The number of procedures per patient was 1 in 23 patients (63.9%), 2 in 11 patients (30.6%), 5 in 1 patient (2.8%), and 7 in 1 patient (2.8%). After consolidating repeated procedures according to the pre-defined criteria, 53 index cases were identified for the final analysis. Furthermore, as mortality was assessed on a per-hospitalization basis, these cases were categorized into 40 distinct hospitalizations to determine in-hospital mortality rates (Figure 1). The majority were male (*n* = 31, 86.1%), and the mean (SD) patient age was 60.9 (12.4) years. The most frequently implanted VAD was HM 3 (*n* = 20, 55.6%), followed by HVAD (*n* = 15, 41.7%) and HM II (*n* = 1, 2.8%). The median interval (IQR) from LVAD implantation to non-cardiac surgery was 100 days [41, 245], and 32 (60.4%) procedures were performed within 180 days of implantation. Most procedures (*n* = 47, 88.7%) were performed on an inpatient basis, and 13 (24.5%) cases were classified as non-elective surgeries. Based on the major surgery classification [5], 13 (24.5%) cases were categorized as major procedures.

General anesthesia was the most used anesthetic technique (*n* = 46, 86.8%). Cardiac anesthesiologists were involved in 13 (24.5%) cases. Arterial line monitoring was utilized in 14 (26.4%) cases, with significantly higher use in major surgeries (*n* = 10, 76.9%) compared to minor surgeries (*n* = 4, 10%) (Fisher’s exact test, *p* < 0.01). Intraoperative hypotension occurred in 24 (45.3%) cases and was observed in specific clinical contexts, including after anesthesia induction, during intrathoracic CO_2_ insufflation for video-assisted thoracoscopic surgery, during episodes of massive bleeding in neurosurgery, under septic conditions in abdominal surgery, and, rarely, during episodes of ventricular arrhythmia. Intraoperative continuous infusion of cardiovascular drugs was required in 18 (33.9%) cases, and 15 (28.3%) required transfusion (Table 1). General surgery was the most performed procedure (*n* = 19, 35.8%), and detailed descriptions of the specific procedures are provided in Supplementary Table S1.

### 3.1. In-Hospital All-Cause Mortality

**Figure 2:**
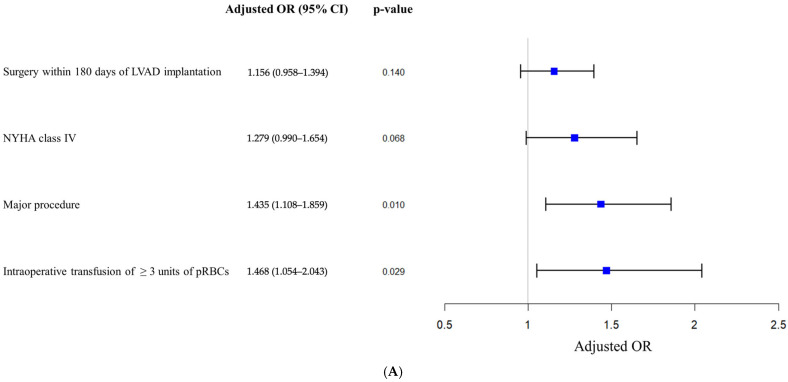

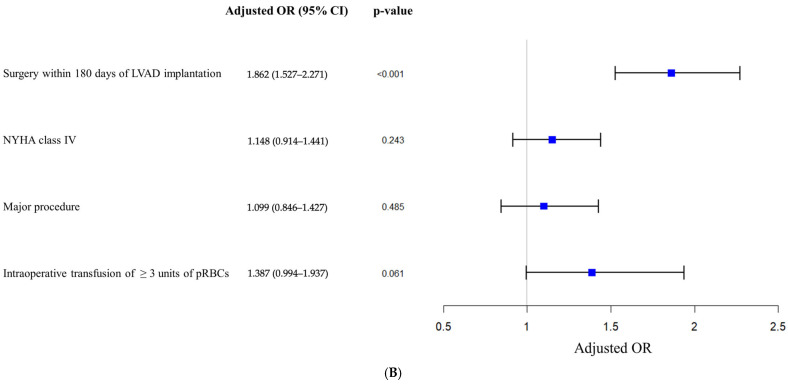
Predictors of in-hospital mortality and postoperative complications in LVAD patients undergoing non-cardiac surgery. Forest plots of the multivariable logistic regression analysis illustrating factors associated with in-hospital mortality (*n* = 40) (**A**), and factors associated with postoperative complications (*n* = 53) (**B**). Adjusted odds ratios (ORs) and 95% CIs are shown. CI, Confidence interval; LVAD, Left ventricular assist device; NYHA, New York Heart Association; OR, Odds ratio; PRBC, Packed red blood cell.

In-hospital mortality, assessed on a per-hospitalization basis, was observed in 8 out of 40 hospitalizations (20%). Detailed information for the mortality cases is described in Table 2. Among patients who died after surgery, six underwent exploratory laparotomy or craniotomy, both classified as major surgeries. Univariable analysis identified preoperative NYHA class IV, hepatopathy, major procedures, non-elective surgery, neurosurgical procedures, higher intraoperative vasoactive–inotropic scores (VIS), estimated blood loss > 500 mL, and need of transfusion as predictors of in-hospital mortality (Table 3). However, after adjusting for confounders, undergoing a major operation (adjusted odds ratio [aOR] 1.44; 95% CI 1.11–1.86; *p* = 0.01) and intraoperative transfusion of ≥ 3 units of packed red blood cell (pRBC) (aOR 1.47; 95% CI 1.05–2.04; *p* = 0.029) remained significantly associated with in-hospital mortality (Figure 2A). Although not statistically significant, NYHA class IV prior to NCS showed a trend toward higher mortality (aOR 1.28; 95% CI 0.99–1.65; *p* = 0.068).

### 3.2. Composite Postoperative Complications

Composite postoperative complications occurred in 32 of the 53 index cases (60.4%) (Table 2). After adjusting for confounders, undergoing NCS within 180 days of LVAD implantation was independently associated with an increased risk of these complications (aOR 1.86; 95% CI 1.53–2.27; *p* < 0.001) (Table 4 and Figure 2B). As detailed in the analysis in Table 5, surgeries performed within 180 days of LVAD implantation were associated with a significantly higher incidence of intraoperative hypotension (area under the curve for MBP < 60 mmHg), elevated intraoperative vasoactive–inotropic scores (VIS), postoperative hypotension requiring clinical intervention, postoperative hemorrhage within 72 h, and increased postoperative transfusion requirements.

### 3.3. Intraoperative Hypotension and Postoperative Outcome

Intraoperative hypotension occurred in 24 (45.3%) of the 53 index cases, involving 19 of 36 individual patients. A higher value for the area for MBP <60 mmHg was significantly associated with in-hospital mortality (OR, 1.0007; 95% CI, 1.0004–1.0010; *p* < 0.001) and with the occurrence of composite postoperative outcomes (OR, 1.0003; 95% CI, 1.0000–1.0006; *p* < 0.05). A TWA-MBP below 60 mmHg was also related to increased in-hospital mortality (OR, 1.0537; 95% CI, 1.0206–1.0879; *p* < 0.05) (Supplementary Table S2).

## 4. Discussion

Although the LVAD population represents a relatively rare cohort, these patients are physiologically distinct and increasingly encountered in contemporary operating rooms, undergoing a wide spectrum of non-cardiac surgical procedures. Accordingly, the purpose of this study was not to derive procedure-specific prognostic estimates, but to characterize perioperative clinical courses and identify vulnerability patterns in LVAD patients across diverse non-cardiac surgical settings using clinically meaningful stratification frameworks. Therefore, the identified associations should be interpreted as indicators of perioperative vulnerability in LVAD patients rather than predictors of outcomes for specific surgical procedures.

In this single-center retrospective study, we analyzed 53 non-cardiac surgeries performed in 36 patients with LVADs, of whom 97% were supported by third-generation centrifugal-flow devices (HeartMate 3 or HVAD). The in-hospital mortality rate was 20%, and postoperative complications occurred in 60.4% of cases. Major surgery (aOR 1.44), transfusion of ≥ 3 units of packed RBCs (aOR 1.47), and surgery within 180 days of LVAD implantation (aOR 1.86) were significantly associated with worse outcomes. Furthermore, patients who remained in NYHA class IV at the time of surgery showed a trend toward increased mortality, although this was not statistically significant. All these NYHA class IV patients had previously required ECMO, but prior ECMO support alone was not independently associated with adverse events, potentially reflecting functional recovery after LVAD implantation. These findings highlight the need for meticulous perioperative planning and individualized risk evaluation, particularly in patients undergoing early surgery or with persistent functional impairment.

While prior studies included endoscopic procedures that did not involve anesthesia [7,10], our analysis focused on patients who underwent surgeries under anesthetic management, predominantly under general anesthesia. Among the eight cases of in-hospital mortality, six occurred in patients who had undergone major surgery: three neurosurgical procedures due to intracranial hemorrhage and three exploratory laparotomies for bowel ischemia. Notably, all three patients who underwent exploratory laparotomy experienced in-hospital mortality. Among the four patients who underwent neurosurgery, three resulted in mortality. In addition, intraoperative blood loss exceeding 500 mL or the need for pRBC transfusion was associated with an increased risk of in-hospital mortality in our univariable analysis. After adjusting for confounders, transfusion of more than three units of pRBC remained significantly associated with increased mortality (OR, 1.468; 95% CI 1.054–2.043; *p* = 0.029), supporting the previously established association between intraoperative pRBC transfusion and postoperative mortality and morbidity [11,12,13]. Given the unique physiology of LVAD patients, the risks associated with high-volume transfusion are particularly pronounced. Rapid volume expansion can lead to acute right ventricular (RV) distention and a subsequent leftward shift in the interventricular septum, potentially triggering suction events or acute RV failure [14]. Furthermore, transfusion-related immunomodulation can exacerbate pre-existing device-related coagulopathy [15]. Therefore, our results suggest that implementing a restrictive transfusion strategy, along with meticulous surgical hemostasis to limit transfusion volume to less than 3 units may be crucial for improving the prognosis of LVAD patients undergoing NCS.

During our retrospective data collection and analysis, we identified a lack of consistent documentation regarding intraoperative LVAD parameters. Despite continuous LVAD parameter monitoring via the console by LVAD coordinators in all cases requiring general anesthesia, these were only sporadically recorded in the anesthesia records. As a result, it was not feasible to analyze LVAD flow or pulsatility index (PI) at the time of intraoperative hypotensive events. Instead, we analyzed the association between postoperative outcomes and the available intraoperative data, including recorded blood pressure and the use of cardiovascular drugs. Higher intraoperative VIS and greater hypotension burden measured by total intraoperative area for MBP < 60 mmHg were associated with adverse postoperative outcomes. For patients with LVAD, the recommended MBP range is 70–85 mmHg, with MBP below 60 mmHg avoided to prevent organ hypoperfusion [16,17]. In the present study, MBP < 60 mmHg was used to define a lower boundary for potential hypoperfusion, whereas the severity of hypotension was evaluated using cumulative burden metrics, allowing assessment of sustained exposure below this threshold while minimizing the influence of brief, clinically insignificant blood pressure fluctuations. In our analysis, a TWA-MBP below 60 mmHg was significantly associated with in-hospital mortality but not with composite outcomes. However, the total area below this threshold was associated with both endpoints, underscoring that total hypotensive exposure—integrating depth and duration—may better predict adverse outcomes in LVAD patients.

Another interesting finding of our study was the association between early surgery performed within 180 days after LVAD implantation and unfavorable postoperative outcomes. The cardiovascular system undergoes progressive adaptation following LVAD implantation, with initial adjustments occurring within days, more substantial remodeling and stabilization over 3–6 months, and full hemodynamic and neurohormonal adaptation typically achieved by 6–12 months [18,19,20]. Surgical procedures performed during this early period of incomplete adaptation may predispose patients to intraoperative hemodynamic instability. Mentias et al. reported that performing NCS within six months of LVAD implantation was associated with a higher incidence of major adverse cardiovascular events (MACE), including in-hospital or 30-day mortality, ischemic stroke, and intracerebral hemorrhage [2]. Similarly, Mathis et al. observed that cases complicated by postoperative AKI had significantly shorter LVAD support duration at the time of surgery compared to cases without AKI (median 84 days vs. 390 days; *p* < 0.001) [6]. Our analysis, presented in Table 5, further underscores this vulnerability. Undergoing NCS within 180 days of LVAD implantation was associated with significantly higher rates of intraoperative and postoperative hypotension, increased intraoperative VIS, and postoperative bleeding.

Among patients who remained in NYHA class IV prior to undergoing non-cardiac surgery after LVAD implantation, there was a trend toward higher postoperative mortality, although it did not reach statistical significance (*p* = 0.068). Notably, all these patients had required ECMO support prior to LVAD implantation. However, a history of ECMO support itself was not significantly associated with intraoperative hypotension or postoperative complications following non-cardiac surgery. This may reflect the fact that many patients with prior ECMO support experienced functional improvement after LVAD implantation, resulting in a distribution of preoperative NYHA classes ranging from II to IV. This observation aligns with previous reports indicating that a substantial proportion of patients show improvement in NYHA class after LVAD implantation [21,22,23,24]. Nonetheless, due to the small sample size, larger studies are needed to better understand the impact of prior ECMO use and persistent advanced NYHA class on postoperative outcomes after noncardiac surgery.

Several perioperative risk factors identified in this study, including surgical severity, bleeding or transfusion burden, and perioperative hypotension, are not unique to patients with LVADs and have also been reported in general surgical populations. However, patients supported with LVADs constitute a physiologically distinct group characterized by altered preload–afterload relationships, limited cardiovascular reserve, and reduced capacity to compensate for acute hemodynamic stress. Within this context, perioperative stressors that may be transient or clinically tolerable in non-LVAD patients can result in disproportionately adverse clinical consequences.

The present study therefore does not suggest that LVAD patients experience fundamentally different types of perioperative complications but rather demonstrates how commonly encountered perioperative stressors manifest within a uniquely vulnerable physiological framework. Notably, the association between early non-cardiac surgery after LVAD implantation and unfavorable postoperative courses highlights a temporally defined period of increased vulnerability, during which incomplete hemodynamic adaptation may predispose patients to adverse outcomes. Taken together, these findings underscore the importance of LVAD-specific perioperative risk awareness and tailored management strategies.

This study has several limitations. First, as this was a single-center retrospective study, the total number of index cases (*n* = 53) and the small number of in-hospital mortality events (*n* = 8) limited the statistical power of our multivariable analysis. Due to being underpowered, our findings regarding surgical timing and transfusion thresholds should be considered exploratory in nature. These results require further validation in larger, prospective, or multi-center cohorts before they can be definitively integrated into clinical practice. In addition, this limitation was evident in our inability to assess clinically meaningful associations for certain variables. For instance, while three out of four neurosurgical procedures for intracranial or subdural hemorrhage resulted in mortality, our univariable regression analysis did not identify neurosurgery as a significant risk factor due to the insufficient sample size. Similarly, although female sex has been reported as a risk factor for perioperative MACEs in previous studies [2], gender-based comparisons were not feasible in our study as only five surgeries were performed in female patients. Nonetheless, the use of single-center electronic medical records enabled highly reliable data extraction through rigorous and repeated reviews. Second, intraoperative LVAD parameters were documented in only 8 of 53 cases. Although we analyzed the available medical records on vasopressor use and blood pressure monitoring, the absence of simultaneously recorded LVAD parameters limited our ability to comprehensively evaluate device performance and its relationship to intraoperative hemodynamic events. Implementing a standardized protocol for intraoperative LVAD parameter documentation is warranted to enable more thorough assessment of hemodynamic status during surgery. Third, preoperative echocardiographic data were collected for all patients; however, the timing of the examinations varied considerably. In some cases, the most recent echocardiogram was performed prior to LVAD implantation, whereas in others, it was conducted after implantation but several months before non-cardiac surgery. Although the importance of RV function in LVAD patients is well established [25], its assessment via echocardiography was not standardized. Consequently, only partial data were available for some patients. Specifically, RV fractional area change (RVFAC) was measured in only 8 of 53 cases, and tricuspid annular plane systolic excursion (TAPSE) was recorded in 29 cases. This variability in echocardiographic assessment limited our ability to accurately evaluate preoperative RV function.

## 5. Conclusions

In this retrospective study of 53 non-cardiac surgeries in LVAD patients, most of whom were supported by third-generation centrifugal pumps, major surgery, early surgical timing, and high transfusion volume were associated with worse outcomes. Patients with NYHA class IV showed a trend toward higher mortality, often with a prior ECMO history, although ECMO itself was not an independent risk factor. These findings indicate the need for individualized perioperative planning in this high-risk group.

## Figures and Tables

**Table 1:** Patient characteristics and perioperative context.

Baseline Characteristics	
Age	60.9 ± 12.4
Male sex	48 (90.6)
Body Mass Index (BMI)	21.0 ± 2.8
Comorbidities	
Hypertension	19 (35.8)
Arrhythmia	25 (47.2)
Diabetes mellitus	16 (30.2)
Acute kidney injury	14 (26.4)
Chronic kidney disease	26 (49.1)
Cerebrovascular accident	9 (17.0)
Hepatopathy	6 (11.3)
Previous ECMO history prior to LVAD implantation	37 (69.8)
NYHA functional class	
2	11 (20.8)
3	29 (54.7)
4	13 (24.5)
LVAD type	
HVAD	27 (50.9)
HeartMate 3	24 (45.3)
HeartMate 2	2 (3.8)
Interval from LVAD implantation to NCS (days)	100 [41, 245]
Post-LVAD NCS ≤ 180 days	32 (60.4)
Implantable cardioverter–defibrillator	18 (34.0)
Preoperative evaluation	
Hemoglobin (g/dL)	10.1 ± 1.6
INR	1.7 ± 0.5
Prognostic nutritional index	3.6 ± 0.6
Pulmonary edema on CXR	16 (30.2)
Pleural effusion on CXR	21 (39.6)
Intraoperative characteristics	
Non-elective surgery	13 (24.5)
Department	
General surgery ^†^	19 (35.8)
Thoracic surgery	13 (24.5)
Neurosurgery	4 (7.5)
Orthopedic surgery	4 (7.5)
Plastic surgery	7 (13.2)
Head and neck surgery	3 (5.7)
Genitourinary surgery	3 (5.7)
Major procedure	13 (24.5)
Type of anesthesia	
General	46 (86.8)
MAC	6 (11.3)
Regional block	1 (1.9)
Cardiac anesthesiologist	13 (24.5)
VAD nurse specialist	46 (86.8)
Anesthetic duration (min)	111 [75, 176]
Arterial line monitoring	14 (26.4)
Central venous line monitoring	13 (24.5)
Intraoperative hypotension	24 (45.3)
Vasoactive–inotropic score	0 [0, 4.5]
Estimated blood loss ≥ 500 mL	9 (17.0)
Intraoperative transfusion	15 (28.3)
Packed red blood cells	28 (52.8)
Fresh frozen plasma	10 (18.9)
Single-donor platelets	5 (9.4)
Cryoprecipitate	0 (0.0)
Discharge	
PACU	24 (45.3)
ICU	29 (54.7)

Data expressed as mean (±SD), median [IQR], or *n* (%). CXR, Chest X-ray; ICU, Intensive care unit; INR, International normalized ratio; IQR, Interquartile range; ECMO, Extracorporeal membrane oxygenation; LVAD, Left ventricular assist device; MAC, Monitored anesthesia care; NYHA, New York Heart Association; PACU, Post-anesthesia care unit; SD, Standard deviation; VAD, Ventricular assist device. ^†^ General surgery included procedures such as exploratory laparotomy, cholecystectomy, and appendectomy. Detailed descriptions of all specific procedures are available in Supplementary Table S1.

**Table 2:** Postoperative Outcomes.

Postoperative Outcomes	
In-hospital all-cause mortality	8 (20)
s/p exploratory laparotomy	3
s/p craniotomy for hematoma removal	3
s/p revision tracheostomy d/t VAP	1
Uncontrolled gastrointestinal bleeding	1
Composite outcomes	32 (60.4)
Acute kidney injury	5 (9.4)
Cerebrovascular accident	3 (5.7)
Postoperative pulmonary complications	6 (11.3)
Postoperative hemorrhage	14 (26.4)
Hypotension	18 (34.0)
Thrombotic events	1 (1.9)
Transfusion	32 (60.4)
Packed red blood cells	28 (52.8)
Fresh frozen plasma	10 (18.9)
Single-donor platelets	7 (13.2)
Cryoprecipitate	1 (1.9)
Hospital length of stay (days)	59 [17, 220]
Postoperative length of stay (days)	25 [8, 94]

Data expressed as median [IQR] or *n* (%). VAP, ventilator-associated pneumonia.

**Table 3:** Predictors of In-hospital Mortality.

Variables	Univariable Analysis OR (95% CI)	*p* Value	Multivariable Analysis OR (95% CI)	*p* Value
Predefined perioperative risk framework variables				
Major surgery	1.826 (1.478–2.254)	<0.001	1.435 (1.108–1.859)	0.010
NCS ≤ 180 days after LVAD	1.238 (0.960–1.595)	0.108	1.156 (0.958–1.394)	0.140
EBL ≥ 500 mL	1.569 (1.159–2.123)	0.006		
Transfusion ≥ 3 pRBC	1.985 (1.446–2.725)	<0.001	1.468 (1.054–2.043)	0.029
Vasoactive–inotropic score	1.022 (1.013–1.031)	<0.001		
Patient-related clinical modifiers				
NYHA functional class	1.314 (1.107–1.559)	0.003		
NYHA class IV	1.569 (1.159–2.123)	0.006	1.279 (0.990–1.654)	0.068
Hepatopathy	1.423 (1.015–1.996)	0.048		
Diabetes mellitus	0.744 (0.576–0.959)	0.028		
Preoperative INR	1.569 (1.280–1.923)	<0.001		
Procedural descriptors (exploratory)				
Surgical Department				
General surgery ^†^	1.063 (0.823–1.374)	0.642		
Thoracic surgery	0.796 (0.545–1.161)	0.243		
Neurosurgery	1.843 (1.264–2.686)	0.003		
Cardiac anesthesiologist	0.892 (0.659–1.206)	0.462		
LVAD nurse attendance	1.265 (0.893–1.792)	0.193		

Logistic regression analysis was performed based on 40 hospitalizations of LVAD patients who underwent non-cardiac surgery. CI, Confidence interval; EBL, Estimated blood loss; INR, International normalized ratio; LVAD, Left ventricular assist device; NCS, Non-cardiac surgery; NYHA, New York Heart Association; OR, Odds ratio; PRBC, Packed red blood cell. ^†^ General surgery included procedures such as exploratory laparotomy, cholecystectomy, and appendectomy. Detailed descriptions of all specific procedures are available in Supplementary Table S1.

**Table 4:** Predictors of Composite Postoperative Complications (*n* = 53).

Variables	Univariable Analysis OR (95% CI)	*p* Value	Multivariable Analysis OR (95% CI)	*p* Value
Predefined perioperative risk framework variables				
Major surgery	1.379 (1.022–1.860)	0.040	1.099 (0.846–1.427)	0.485
NCS ≤ 180 days after LVAD	1.983 (1.623–2.422)	<0.001	1.862 (1.527–2.271)	<0.001
EBL ≥ 500 mL	1.612 (1.156–2.248)	0.007		
Transfusion ≥ 3 pRBC	1.579 (1.084–2.300)	0.021	1.387 (0.994–1.937)	0.061
Vasoactive–inotropic score	1.028 (1.011–1.044)	0.002		
Patient-related clinical modifiers				
NYHA functional class	1.274 (1.055–1.538)	0.015		
NYHA class IV	1.379 (1.022–1.860)	0.040	1.148 (0.914–1.441)	0.243
Prognostic nutritional index	0.740 (0.604–0.906)	0.005		
Procedural descriptors (exploratory)				
Surgical Department				
Plastic surgery	1.579 (1.084–2.300)	0.021		
Thoracic surgery	0.748 (0.553–1.011)	0.065		
Non-elective surgery	1.016 (0.743–1.387)	0.923		
Cardiac anesthesiologist	1.125 (0.825–1.534)	0.462		
LVAD nurse attendance	1.443 (0.983–2.117)	0.067		

Logistic regression analysis was performed based on 53 index cases of LVAD patients who underwent non-cardiac surgery. CI, Confidence interval; EBL, Estimated blood loss; INR, International normalized ratio; LVAD, Left ventricular assist device; NCS, Non-cardiac surgery; NYHA, New York Heart Association; OR, Odds ratio; PRBC, Packed red blood cell.

**Table 5:** Perioperative Characteristics and Postoperative Outcomes in Patients Undergoing Noncardiac Surgery within 180 Days after LVAD Implantation.

	Odds Ratio	*p* Value
Preoperative evaluation		
NYHA class IV	4.842	0.053
Previous ECMO prior to LVAD	0.043	0.835
INR	0.792	0.432
Non-elective surgery	0.307	0.579
Major surgery	1.648	0.528
Surgical Department		
General surgery ^†^	3.658	0.0463
Thoracic surgery	0.197	0.0209
Neurosurgery	0.639	1
Plastic surgery		0.0339
Intraoperative variables		
Intraoperative transfusion	3.518	0.0665
Intraoperative transfusion of ≥ 3 pRBC	1.742	0.6897
Intraoperative EBL ≥ 500 mL	2.615	0.291
Intraoperative hypotension		
Hypotensive event	6.803	0.002
Area for MBP < 60 mmHg (mmHg × min)		0.012
TWA-MBP < 60 mmHg (mmHg)		0.0798
VIS		0.004
Postoperative outcome		
AKI		0.144
PPC		0.0702
CVA	1.326	1
Postoperative hemorrhage	5.532	0.0239
Postoperative hypotension	9.119	0.0028
Postoperative transfusion	14.708	0.0001
LVAD related adverse event	7.617	0.0058

Association between categorical variables was assessed using Fisher’s exact test or Chi-squared test, as appropriate. *p*-values for continuous variables were derived from independent *t*-tests comparing patients undergoing surgery within versus after 180 days of LVAD implantation. AKI, Acute kidney injury; CVA, Cerebrovascular accident; EBL, Estimated blood loss; INR, International normalized ratio; PRBC, Packed red blood cells; LVAD, Left ventricular assist device; NCS, Non-cardiac surgery; NYHA, New York Heart Association (classification); PPC, Postoperative pulmonary complication; TWA-MBP, Time-weighted average of mean blood pressure; VIS, Vasoactive–inotropic score. ^†^ General surgery included procedures such as exploratory laparotomy, cholecystectomy, and appendectomy. Detailed descriptions of all specific procedures are available in Supplementary Table S1.

## Data Availability

The original contributions presented in the study are included in the article, further inquiries can be directed to the corresponding author.

## Supplementary Materials

The following supporting information can be downloaded at: https://www.mdpi.com/article/10.3390/jcm15051748/s1.
