# Supplementary material for: Perioperative Care and Clinical Outcomes of Patients with Left Ventricular Assist Devices Undergoing Noncardiac Surgery in Korea: A Retrospective Study
Source: J Clin Med. 2026 Feb 25;15(5):1748. doi: 10.3390/jcm15051748 (PMC12985548; doi:10.3390/jcm15051748)
Supplement: Supplementary file 1 [file jcm-15-01748-s001.zip › jcm-4101636-supplementary.pdf]

Supplementary Table S1. Type of Noncardiac Surgery

Surgery category		Overall (n=53)	Urgent (n=13)	Elective (n=40)
General		19	5	14
	Anorectal surgery	3		3
	Arteriovenous fistula formation	3		3
	Exploratory laparotomy	3	2	1
	Herniorrhaphy	2		2
	Laparoscopic adhesiolysis	1	1	
	Laparoscopic appendectomy	1	1	
	Laparoscopic cholecystectomy	4	1	3
	Superficial femoral artery thrombectomy	1		1
	Upper abdomen wound repair	1		1
Thoracic		13	3	10
	LVAD drive line site incision and drainage	4	2	2
	Permanent pacemaker wound repair	1		1
	Sternal wound repair	6	1	5
	Video-Assisted Thoracoscopic hematoma evacuation	1		1
	Video-Assisted Thoracoscopic wedge resection	1		1
Neurosurgery		4	3	1
	Craniectomy	1	1	
	Craniotomy	3	2	1
Orthopedic		4	0	4
	Arthroscopic synovectomy, knee	1		1
	Below knee amputation	1		1
	Finger amputation	1		1
	Transmetatarsal and finger amputation	1		1
Head and neck		3	2	1
	Laryngeal microsurgery	1		1
	Revision tracheostomy	2	2	
Plastic		7	0	7
	Debridement and free flap	2		2
	Debridement and local flap	4		4
	Debridement and split-thickness skin graft	1		1
Genitourinary		3	0	3
	Hysteroscopic myomectomy	1		1
	Transurethral resection of the bladder	2		2

Supplementary Table S2. Intraoperative Hypotension and Postoperative Outcome

	Mean (SD), n=28	Median (IQR), n=28	Univariable analysis OR (95% CI)	P value
TWA-MBP, mmHg	73.491 (11.655)	71.519 (64.590, 78.572)		
Monitoring time, min	151.25 (96.000)	130 (87.5, 181.25)		
Hypotension severity (MBP < 60mmHg)				
Area for MBP < 60mmHg, mmHg×min	368.661 (545.066)	145 (15, 473.75)		
In-hospital mortality			1.001 (1.000, 1.001)	<0.001
Postoperative complications			1.000 (1.000, 1.001)	0.034
TWA-MBP < 60mmHg, mmHg	3.943 (3.585)	3.570 (0.75, 5.715)		
In-hospital mortality			1.054 (1.021, 1.088)	0.003
Postoperative complications			1.021 (0.980, 1.064)	0.317

† *The analysis of hypotension severity (Area under the curve and TWA-MBP) was performed on 28 of the 53 index cases that exhibited recorded hypotensive burden (AUC > 0) in the automated anesthesia data system. Clinically significant hypotension was identified in 24 of these cases.* CI, Confidence interval; IQR, Interquartile range; MBP, Mean blood pressure; OR, Odds ratio; SD, Standard deviation.
